# Deep learning in structural and functional lung image analysis

**DOI:** 10.1259/bjr.20201107

**Published:** 2021-04-20

**Authors:** Joshua R Astley, Jim M Wild, Bilal A Tahir

**Affiliations:** 1 POLARIS, Department of Infection, Immunity & Cardiovascular Disease, The University of Sheffield, Sheffield, United Kingdom; 2 Department of Oncology and Metabolism, The University of Sheffield, Sheffield, United Kingdom

## Abstract

The recent resurgence of deep learning (DL) has dramatically influenced the medical imaging field. Medical image analysis applications have been at the forefront of DL research efforts applied to multiple diseases and organs, including those of the lungs. The aims of this review are twofold: (i) to briefly overview DL theory as it relates to lung image analysis; (ii) to systematically review the DL research literature relating to the lung image analysis applications of segmentation, reconstruction, registration and synthesis. The review was conducted following the Preferred Reporting Items for Systematic Reviews and Meta-Analyses guidelines. 479 studies were initially identified from the literature search with 82 studies meeting the eligibility criteria. Segmentation was the most common lung image analysis DL application (65.9% of papers reviewed). DL has shown impressive results when applied to segmentation of the whole lung and other pulmonary structures. DL has also shown great potential for applications in image registration, reconstruction and synthesis. However, the majority of published studies have been limited to structural lung imaging with only 12.9% of reviewed studies employing functional lung imaging modalities, thus highlighting significant opportunities for further research in this field. Although the field of DL in lung image analysis is rapidly expanding, concerns over inconsistent validation and evaluation strategies, intersite generalisability, transparency of methodological detail and interpretability need to be addressed before widespread adoption in clinical lung imaging workflow.

## Introduction

Respiratory diseases constitute significant global health challenges; five respiratory diseases are among the most common causes of death. 65 million people suffer from chronic obstructive pulmonary disease (COPD) and 339 million from asthma.^1,2^ There are 1.8 million new lung cancer cases diagnosed annually and 1.6 million deaths worldwide, making it the most common and deadliest cancer on the planet.^3^ Lung imaging is a critical component of respiratory disease diagnosis, treatment planning, monitoring and treatment assessment. Acquiring lung images, processing them and interpreting them clinically are crucial to achieving global reductions in lung-related deaths. Traditionally, the techniques employed to quantitatively analyse these images evolved from the disciplines of computational modelling and image processing; however, in recent years, deep learning (DL) has received significant attention from the lung imaging community.

DL is a subfield of machine learning that employs artificial neural networks with multiple deep or hidden layers. Whilst the fundamental theory was posited several decades ago,^4^ DL gained international interest in 2012 when AlexNet, a type of neural network referred to as a convolutional neural network (CNN), won the ImageNet Large Scale Visual Recognition Challenge. That paper has been cited over 47,000 times and triggered a renaissance in DL research.^5^ Subsequently, CNNs, and DL more generally, began to impact the medical imaging field profoundly. Development of fully convolutional networks such as V-Net and ConvNet demonstrated how deep-layered architectures could provide valuable functions in solving some of the field’s most critical applications, including common image analysis tasks.^6,7^ Increased computational power due to the reduced cost of graphical processing units (GPUs) and publicly available annotated imaging data sets have since led to rapid developments and applications.^8^

This review assesses the current literature on DL’s role in lung image analysis applications, discusses critical limitations for clinical adoption, and sets out a roadmap for future research.

## Theory

### Artificial neural networks

**Figure 1.**
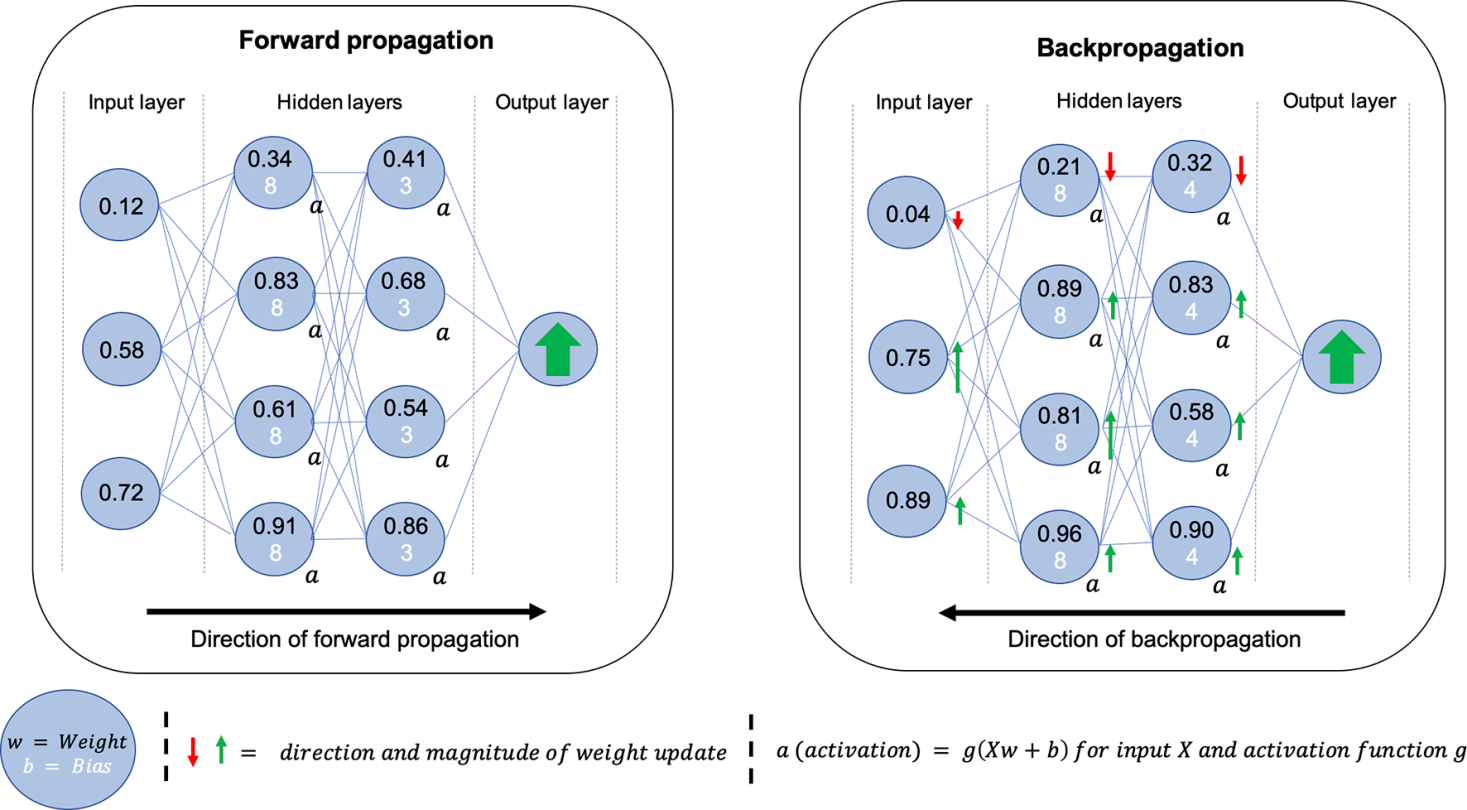
Simplified diagrams of the processes of forward propagation (left) and backpropagation (right) for a neural network with two hidden layers. The neural network is represented as a series of nodes, each of which contains a weight and bias. The weight and bias are combined using the activation function to produce an activation that impacts the strength of connections within the network. Once an input has been passed through the network, it is compared to a desired output, such as an expert segmentation of an anatomical region of interest, to produce a loss. This loss is used to propagate changes to weights and biases, hence, changing the strength of connections for the subsequent example. The continued repetition of this two-step process is known as network training.

**Figure 2.**
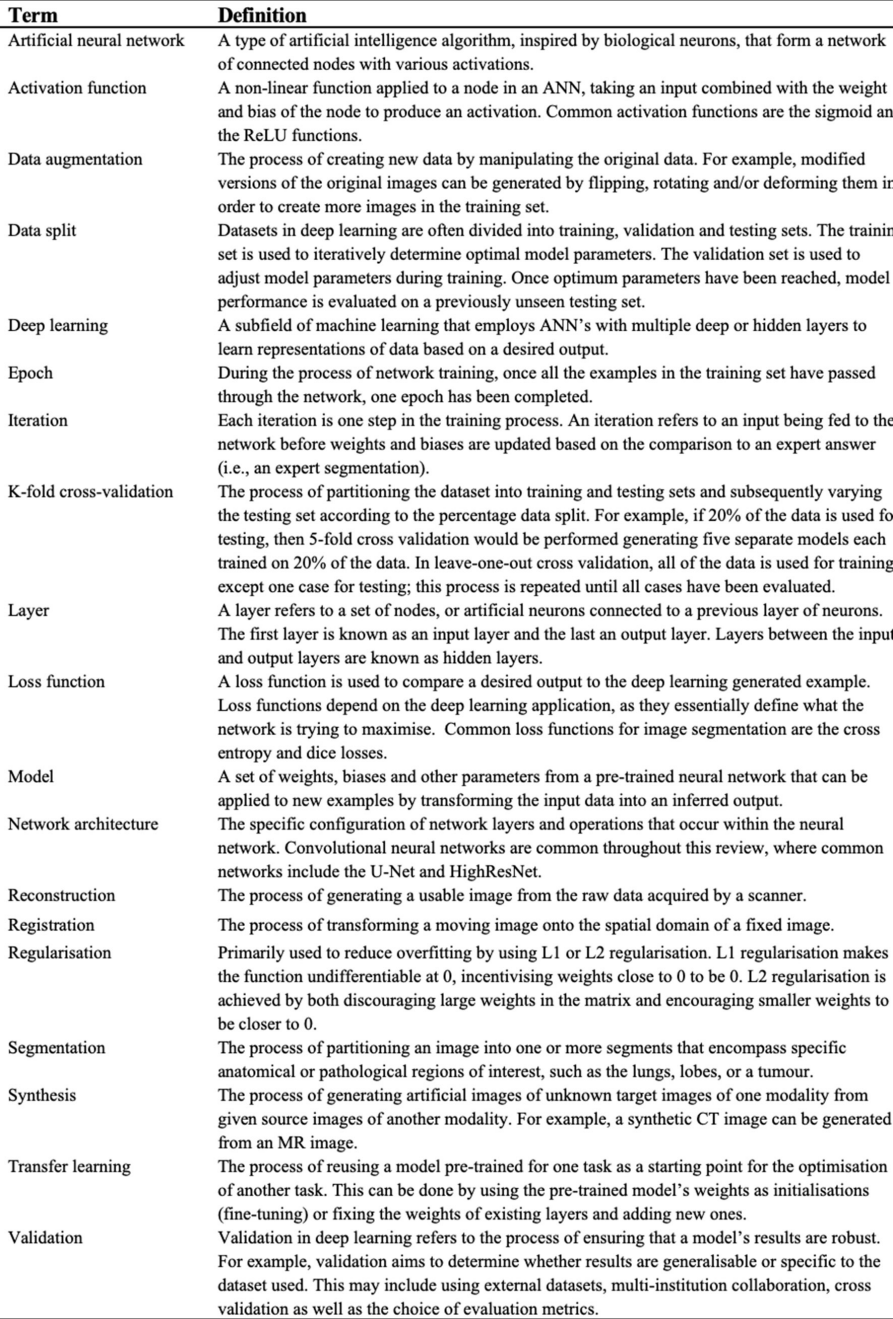
Glossary of key technical terms related to deep learning and image analysis. ANN, artificial neural network.

An artificial neural network (ANN), inspired by biological neurons, can be thought of as a series of connected nodes containing weights and biases which are combined using an activation function to produce an activation; the activation determines the strength of connections within the network. At the heart of DL is optimisation; an ANN learns by optimising weights and biases for a generalisable solution. This optimisation occurs in a two-step process of forward propagation and backpropagation. A basic diagram of an ANN with two hidden layers and generalised examples of forward propagation and backpropagation are shown in Figure 1. The use of hidden layers in the network allows more freedom for the weights and biases to be optimised. Forward propagation refers to the process of feeding an example to the network during training where the output of the neural network is compared to a desired output and a loss is calculated using a loss function. Backpropagation uses this loss to propagate changes in weights and biases throughout the network; thus by continually providing new examples, known as iterations, the model is optimised to approximate the function between the input and output domains. Figure 2 provides a glossary of the key technical terms used in this review.

**Figure 3.**
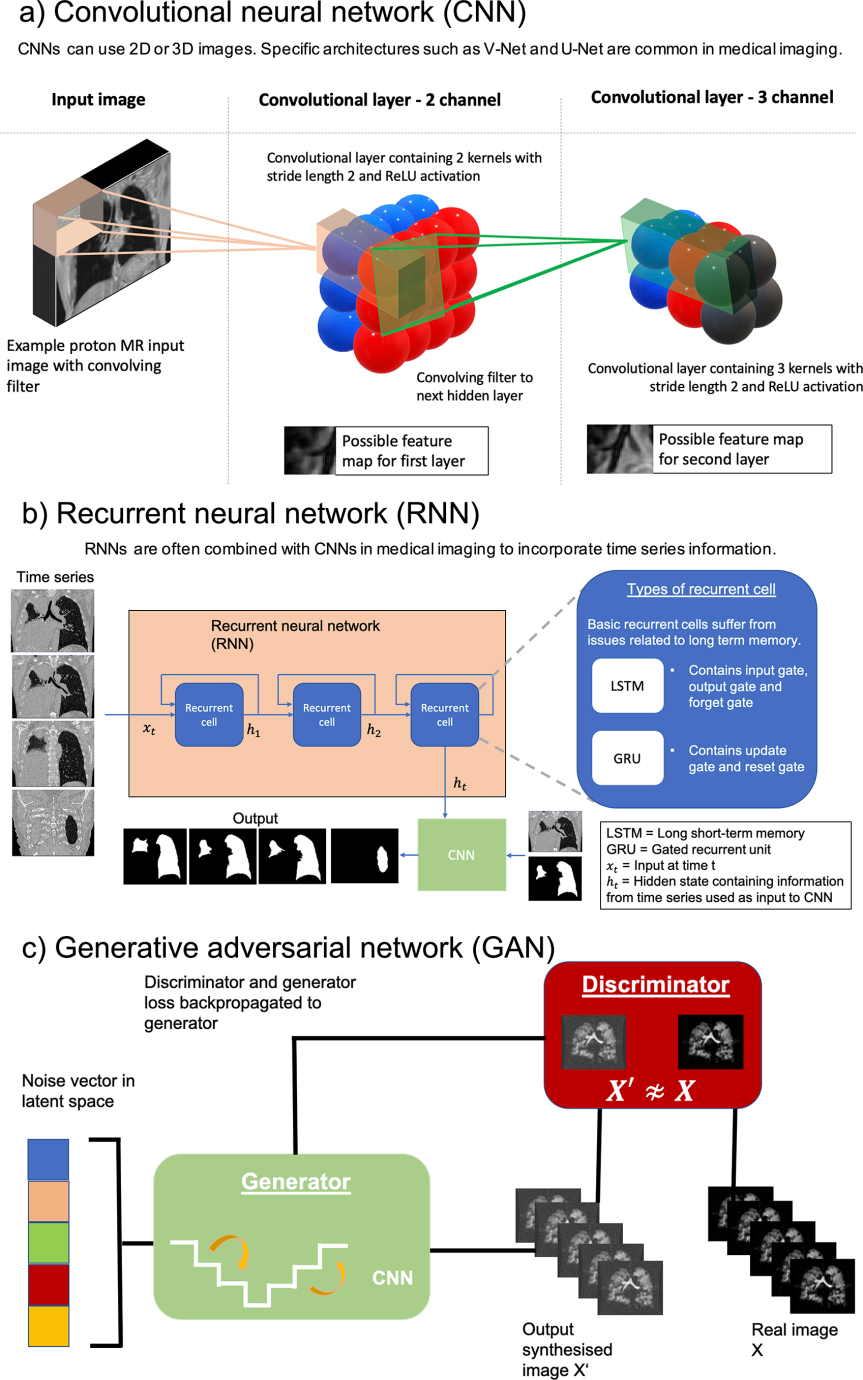
Illustration of three common types of deep learning architectures used in medical imaging: (a) CNN), (**b**) RNN and (c) GAN. In the lung image analysis examples given, the CNN and RNN are used for image segmentation while the GAN is used for image synthesis. CNN, convolutional neural network; GAN, generative adversarial network; RNN, recurrent neural network.

The structure of a DL network is known as an architecture. In the medical imaging field, three key architectures, namely, CNNs, recurrent neural networks (RNNs) and generative adversarial networks (GANs) are particularly prevalent. These structures are outlined in Figure 3. Understanding specific architectures such as V-Nets and GANs requires an in-depth understanding of complex linear algebra and matrix manipulation and is beyond this review’s scope; the interested reader is directed to several excellent papers on the subject.^6,9,10^

### Preprocessing

Before images are fed into a neural network, they are frequently processed, often by accentuating differences between foreground and background voxels, to enhance performance and/or reduce training time. DL theory suggests that in high-dimensional matrices, local minima are very unlikely; instead, saddle points are more common due to the improbable likelihood that every dimension produces a minimum at the same location. These techniques can decrease the likelihood that the algorithm reaches a shallow saddle point, thereby causing slower optimisation. This is achieved through regularisation techniques and limiting outlier intensities. Cropping is regularly used to restrict the processing to voxels within the patient,^11^ or coarse, manually drawn bounding boxes.^12^ Table 1 summarises commonly used preprocessing techniques in the DL lung image analysis literature. In CNNs, other techniques such as batch normalisation, have been shown to reduce training time, acting as secondary regularisation techniques to minimise outliers and improve performance.^62,63^

**Table 1.** Summary of common pre-processing techniques used for lung image analysis tasks, including values prevalent in the literature

*Preprocessing technique*	*Description*	*Modality*	*Literature values*	*References*
*Thresholding*	The process of constraining the pixel values of an image to be between predefined values.	CT, MRI	CT intensity: [-1000, 700 HU] MRI intensity: [0,667]	Wang et al. (2018),^13^ Sousa et al. (2019),^14^ Javaid et al. (2018),^15^ Hofmanninger et al. (2020),^16^ Jiang et al. (2019),^17^ Tahmasebi et al. (2018),^18^ Z. Zhong et al. (2019),^19^ Zhou et al. (2019),^20^ Park et al. (2019),^21^ Gerard et al. (2019),^22^ Yun et al. (2019),^23^ Eppenhof & Pluim (2019),^24^ Fu et al. (2020),^25^ Jiang et al. (2020),^26^ De Vos et al.(2019),^27^ Stergios et al. (2018),^28^ Ren et al. (2019)^29^
*Normalisation and whitening*	The process of transforming the distribution of image pixels to some distribution which is standardised across images.	CT, MRI, X-ray	Normalisation: [0,1] Mean/variance ≈ 0	Wang et al. (2018),^13^ Liu et al. (2019),^30^ Javaid et al. (2018),^15^ Hofmanninger et al. (2020),^16^ Akila Agnes et al. (2018),^31^ Novikov et al. (2018),^32^ Gaal et al. (2020),^33^ Jiang et al. (2019),^17^ Tahmasebi et al. (2018),^18^ Zhou et al. (2019),^20^ Hatamizadeh et al. (2019),^34^ Sandkühler et al. (2019),^35^ Rajchl et al. (2017),^36^ Sentker et al. (2018),^37^ Fletcher and Baltas (2020),^38^ Jiang et al. (2020),^26^ De Vos et al.(2019),^27^ Galib et al. (2019),^39^ Ferrante et al. (2018),^40^ Stergios et al. (2018),^28^ Beaudry et al. (2019),^41^ Duan et al. (2019),^42^ Liu et al. (2020),^43^ Ren et al. (2019),^29^ Olberg et al. (2018)^44^
*Denoising*	The process of removing noise from images in order to improve their quality.	CT, MRI	Gaussian, adaptive patch-based	J.Xu & Liu (2017),^45^ Zha et al. (2019),^46^ Tustison et al. (2019)^47^
*Bias correction*	A technique to correct for the low-frequency bias field that corrupts MR images.	HP gas MRI, MRI	N3/N4 bias correction	Tustison et al. (2019),^47^ Zha et al. (2019),^46^ Rajchl et al. (2017)^36^
*Cropping*	Cropping refers to the process of removing unwanted outer pixels or voxels of an image prior to being inputted to the network. This includes cropping by manually-defined regions of interest or external body masks. Cropping is commonly used to reduce computational cost and/or eliminate the influence of background voxels.	CT, MRI, X-ray, PET	Cropping to body mask, specific organ or manually-defined region.	Negahdar et al. (2018),^12^ Soans & Shackleford (2018),^48^ Zhu et al. (2019),^49^ Hofmanninger et al. (2020),^16^ Zha et al. (2019),^46^ Hooda et al. (2018),^50^ Mittal et al. (2018),^51^ Jiang et al. (2018),^11^ Zhao et al. (2019),^52^ Zhou et al. (2019),^20^ Moriya et al. (2018),^53^ Kalinovsky et al. (2017),^54^ Sandkühler et al. (2019),^35^ Anthimopoulos et al. (2019),^55^ Gao et al. (2016),^56^ Rajchl et al. (2017),^36^ C. Wang et al. (2019),^57^ Juarez et al. (2019),^58^ Juarez et al. (2018),^59^ Eppenhof & Pluim (2019),^24^ Sentker et al. (2018),^37^ Fletcher and Baltas (2020),^38^ Blendowski & Heinrich (2019),^60^ Zhong et al. (2019),^61^ Liu et al. (2020),^43^ Olberg et al. (2018)^44^

HU, Hounsfield unit; PET, Positron emission tomography.

Modalities included are those for which the pre-processing techniques have been used in the reviewed studies. This is not an exhaustive list of pre-processing techniques used.

### Validation

Validation is used to evaluate the performance of trained DL networks and assess their generalisability to non-experimental settings. The goal is to develop a validation strategy that best represents the situation in which the algorithm is to be deployed.

### Evaluation metrics

**Figure 4.**
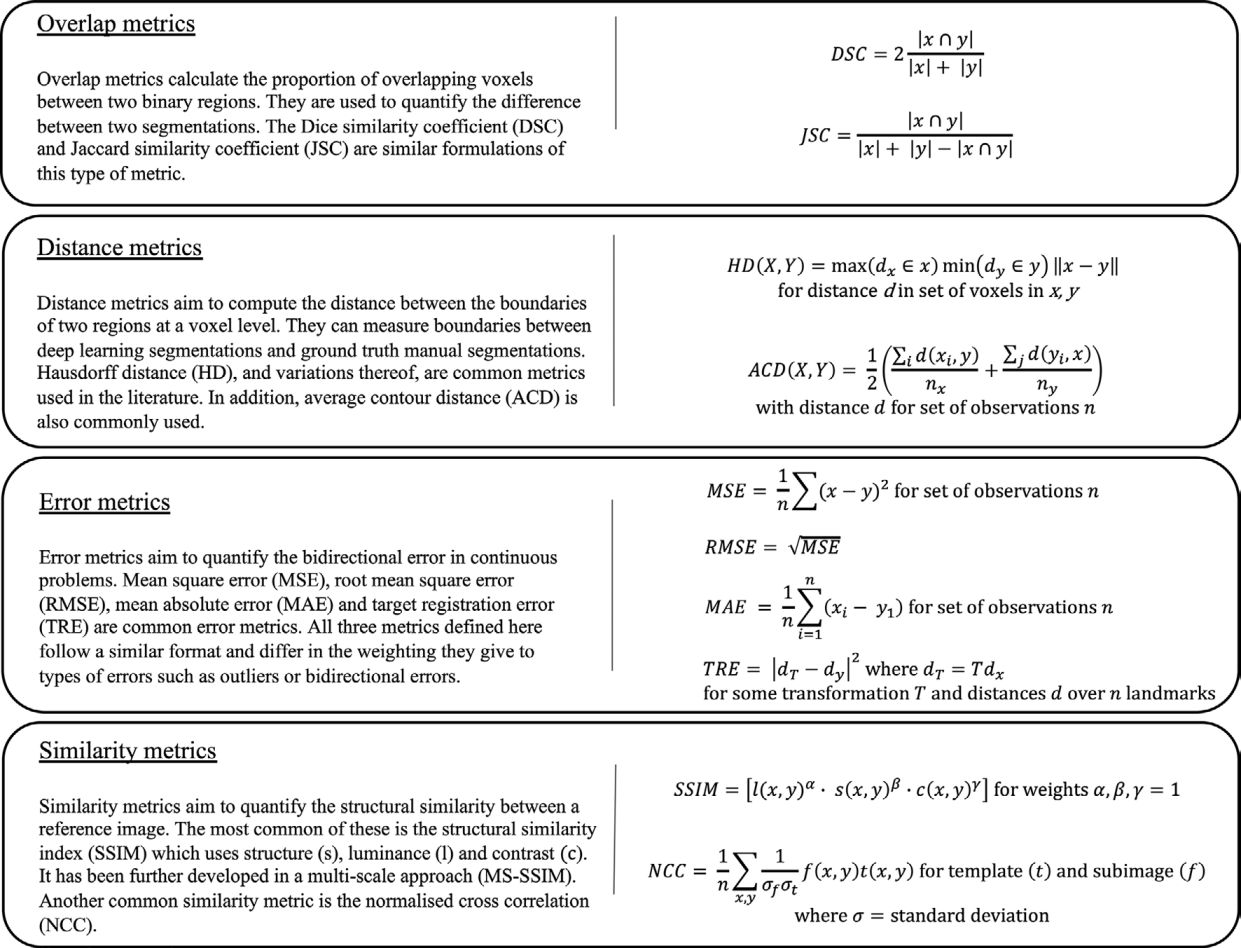
Overview of four key categories of evaluation metrics (overlap, distance, error and similarity) used to evaluate the performance of deep learning methods in medical image analysis. Each category contains brief descriptions and mathematical formulations for some common metrics. In these equations, ‘*x*’ and ‘*y*’ denote the prediction and target of any deep learning task, respectively.

It is imperative to evaluate the performance of DL algorithms accurately. Evaluation metrics can be categorised into overlap, distance, error and similarity metrics and are summarised in Figure 4.

### Validation techniques

Aside from the training set, an internal validation set is commonly used for tuning DL parameters to improve performance. A testing set is then used to provide an unbiased evaluation of performance on unseen data. In this review, validation sets used throughout the training phase are counted as training sets as the network has previously seen these images before testing. Therefore, the data split is the percentage of the total data used for training and internal validation *vs* that used for testing. Maintaining completely separate testing sets is somewhat uncommon in the literature and represents the ideal form of validation.^22,23,64^ Validating on external multicentre data sets that have not been used for training should be the gold-standard in ensuring comparison between methods and generalisability.^65^ However, this is uncommon as single-centre data sets, split into training and testing sets, are frequently used. To make the validation process more robust and generalisable, specific techniques are applied, such as k-fold cross-validation. In fourfold cross-validation, the datas et is randomly partitioned into a 75/25% training/testing split; this process is repeated with four different 25% blocks. Another approach is leave-one-out cross-validation which uses all of the data for training except one case for testing and repeats until all cases have been evaluated.

## Methods

**Figure 5.**
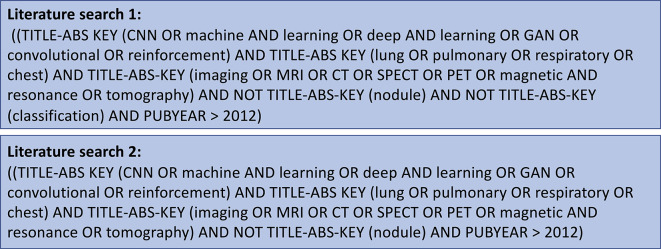
The search strategy used on Scopus, Web of Science and PubMed to identify relevant studies for inclusion in the review. Further studies that met the selection criteria were identified by handsearching references and through the authors’ input.

The protocol for this literature review was performed using the preferred reporting items for systematic reviews and meta-analyses (PRISMA)-statement.^66^ The literature search was conducted on 1 April 2020 using multiple databases (Web of Science, Scopus, PubMed) and aimed to identify studies written in English published between 1 January 2012, the same year that the seminal AlexNet paper was published,^5^ and the date of the search. The search strategy is defined in Figure 5. Further studies that met the selection criteria were identified by handsearching references and through the authors’ input.

Several recent reviews have focussed primarily on DL-based lung classification and detection^67–69^; accordingly, this review was limited in scope to the lung image analysis applications of segmentation, registration, reconstruction and synthesis. Both published peer-reviewed scientific papers and conference proceedings were included due to recent developments in the field.

## Results and discussion

### Study selection

**Figure 6.**
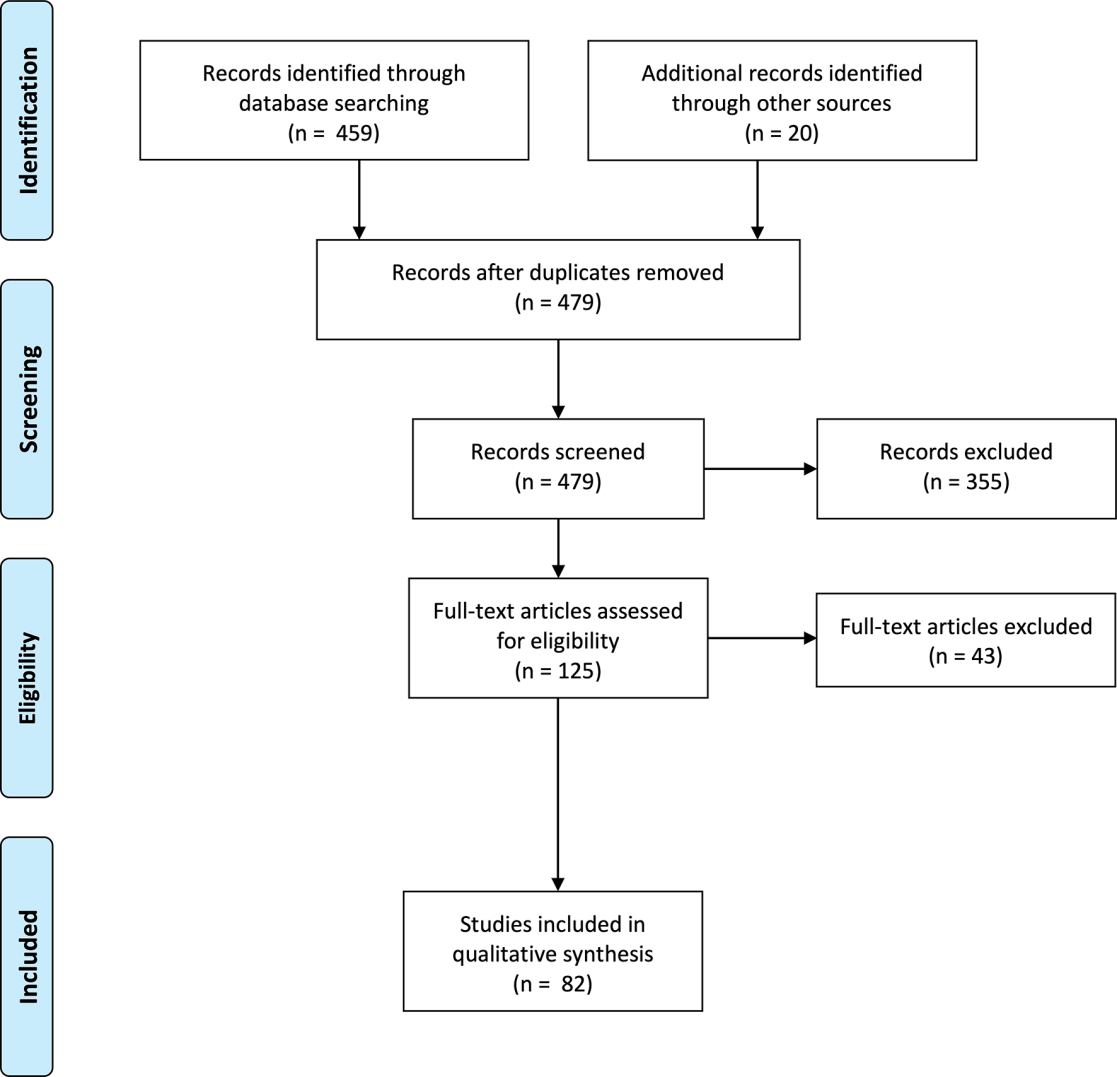
PRISMA flowchart of studies identified, screened, assessed for eligibility and included in the literature review analysis. PRISMA, preferred reporting items for systematic reviews and meta-analyses.

479 non-overlapping papers were retrieved. 355 papers were excluded due to not meeting the eligibility criteria. In particular, many papers focused on classification or used traditional machine learning techniques beyond this review’s scope. Upon reviewing the remaining papers, 82 studies were included for analysis. The PRISMA flowchart is shown in Figure 6.

**Figure 7.**
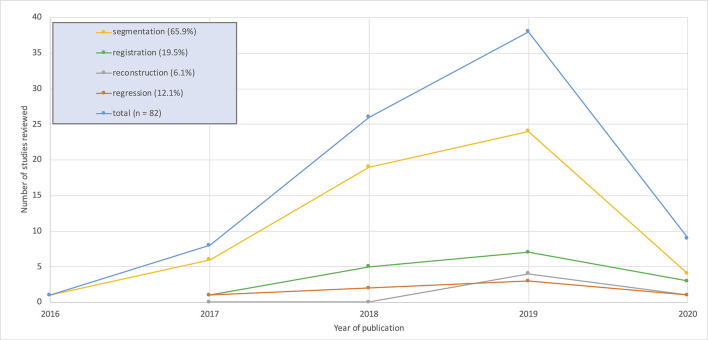
Graphical overview of the number of studies per year for the four image analysis applications considered in this review. 2020 values calculated up to 1 April 2020.

No studies that met the inclusion criteria were published before 2016 with the majority appearing since 2018. Image segmentation applications accounted for 65.9% of the studies reviewed. The remaining 34% are divided between synthesis, reconstruction and registration applications. Full details are shown in Figure 7.

**Figure 8.**
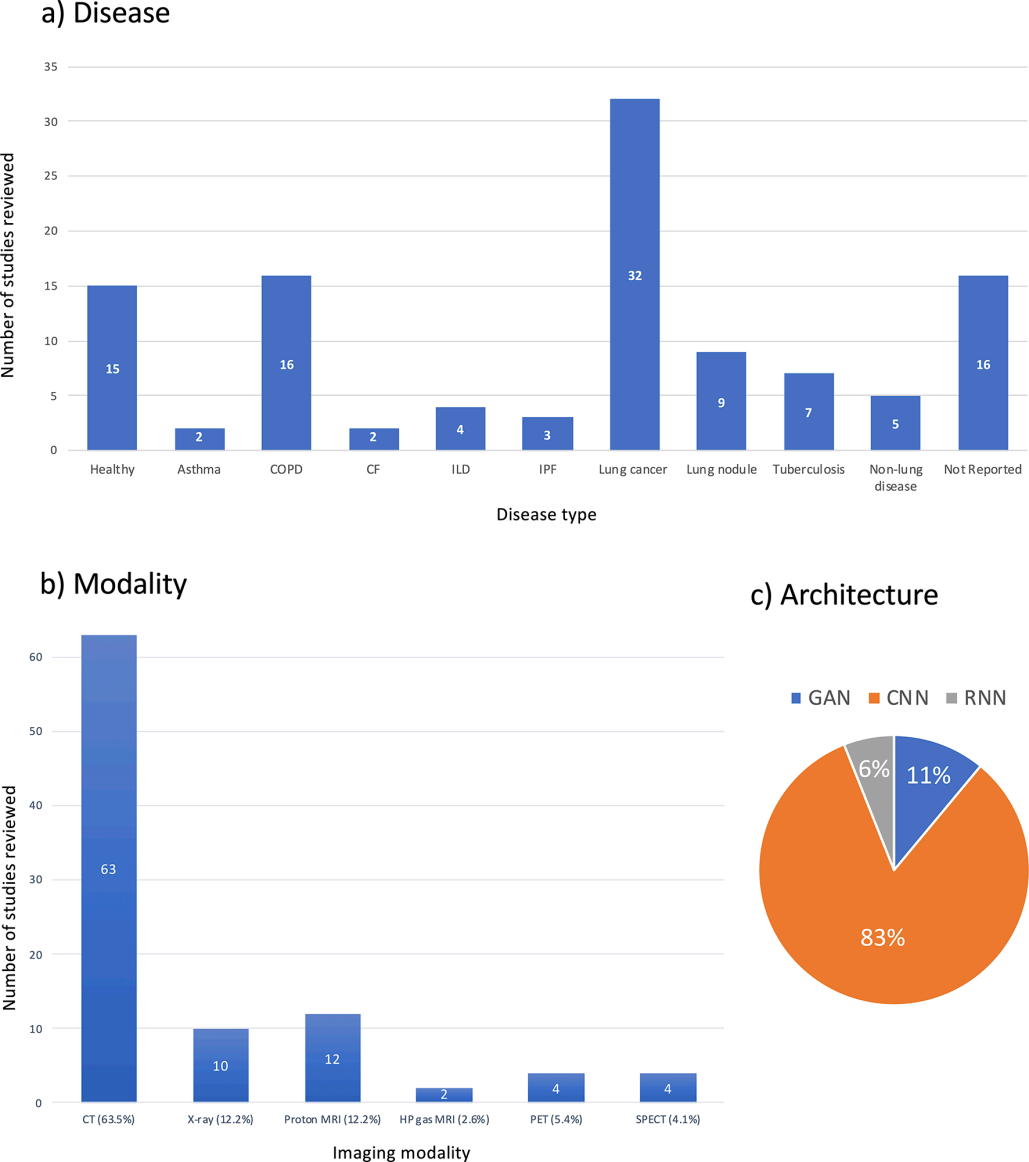
Graphical overview of breakdown of deep learning lung image analysis studies reviewed by (a) disease present in patient cohorts, (**b**) imaging modality and (c) architecture. Absolute numbers of papers are provided in (a, b).

The majority of studies reviewed used structural imaging modalities (87.8%), with most using CT (63.5%). Functional lung imaging studies only constitute 12.1% of the reviewed studies and are spread across PET, SPECT and hyperpolarised gas MRI. Graphical summaries of the studies reviewed with respect to disease present in patient cohorts, imaging modality and architecture are shown in Figure 8.

### Segmentation

Image segmentation is the process of partitioning an image into one or more segments that encompass anatomical or pathological specific regions of interest (ROIs), such as the lungs, lobes, or a tumour. Studies describing DL-based segmentation applications of pulmonary ROIs are summarised in Table 2.

**Table 2.** Summary of reviewed studies on deep learning for lung image segmentation. The entries are arranged alphabetically by pulmonary region of interest (ROI), followed by modality

*Study*	*Modality*	*ROI*	*Disease*	*Number of subjects*	*Dimentionality*	*Architecture*	*Pre-processing*	*Percentage data split* (training*/testing)	*Performance*
*Wang et al. (2018*)^13^	CT	Whole lung	COPD, IPF	575	2D	ResNet-101	Clipped −1000 to +1000 HU, Normalisation [0,1]	5-fold CV	DSC = 0.988 ± 0.012 ASD = 0.562±0.52 mm
*Dong et al. (2019*)^70^	CT	Whole lung	Lung cancer	35	3D	U-Net-GAN		LOOCV	DSC = 0.97±0.01 HD95 = 2.29±2.64 mm MSD = 0.63±0.63 mm
*Liu et al. (2019*)^30^	CT	Whole lung	NR	100	2D	SegNet	Class grouping, Normalisation [−1000,800]	40/60	DSC = 0.98
*Lustberg et al. (2018*)^71^	CT	Whole lung	Lung cancer	470	NR	CNN		95/5	DSC = 0.99±0.01 Median HD = 0.4±0.2 cm
*Negahdar et al. (2018*)^12^	CT	Whole lung	Multiple	83	3D	V-Net	Bounding box for lung, cropped to bounding box	58/42	DSC(*n* = 12)=0.983±0.002 DSC(*n* = 23)=0.990±0.002
*Soans & Shackleford (2018*)^48^	CT	Whole lung	Lung cancer	422	3D	CNN with spatial constraints	ROI extraction for organ localisation	71/29	ROC(Left)=0.954 ROC(right)=0.949
*Soliman et al. (2018*)^72^	CT	Whole lung	NR	95	3D	Deep-CNN	Post-processed hole filling	LOOCV	DSC = 0.984±0.068 HD95 = 2.79±1.32 mm PVD = 3.94±2.11%
*Sousa et al. (2019*)^14^	CT	Whole lung	Lung lesion	908	3D	Modified V-Net	Clipped [−1000, 400 HU]	98/2	ASD = 0.576 mm DSC = 0.987
*X. Zhou et al. (2017*)^73^	CT	Whole lung	NR	106	2D/3D	FCN VGG16	Transfer learning from ImageNet ILSVRC‐2014	95/5	JSC = 0.903±0.037
*Zhu et al. (2019*)^49^	CT	Whole lung	Lung Cancer	66	3D	U-Net	Cropping to ROI	55/45	DSC = 0.95±0.01 MSD = 1.93±0.51 mm HD95 = 7.96±2.57 mm
*Gerard et al. (2018*)^74^	CT	Whole lung	COPD, IPF	1749	3D	Course-Fine ConvNet	Transfer learning from COPDGene and SPIROMICS, fine-tuned on animal model	92/8	JSC = 0.99 ASD = 0.29 mm
*Javaid et al. (2018*)^15^	CT	Whole lung	Lung cancer	13	2D	Dilated U-Net	Only axial slices selected, clipped −1000 to 3000 HU, Normalisation [0,1]	94/6	DSC = 0.99 ± 0.01 HD ≈ 4.5 mm
*J. Xu & Liu (2017*)^45^	CT	Whole lung	NR	20	2D	MFCNN	gaussian denoising	50/50	DSC = 0.754
*Hu et al. (2020*)^75^	CT	Whole lung	NR	75	2D	Mask R-CNN +k-means		NR	DSC = 0.973 ±0.032
*Hofmanninger et al. (2020*)^16^	CT	Whole lung	Multiple	266	2D	U-Net	Body mask, Clipped [−1024, 600 HU], Normalisation [0,1]	87/13	DSC = 0.98 ±0.03 HD95 = 3.14 ±7.4 mm MSD = 0.62 ±0.93
*Xu et al. (2019*)^76^	CT	Whole lung	Lung cancer, COPD	224	2D	one layer CNN	Post-processed hole filling	8-fold CV	DSC = 0.967 ±0.001 HD = 1.44±0.04 mm
*Tustison et al. (2019*)^47^	HP gas MRI Proton MRI	Functional lung Whole lung	NR NR	113 268	2D 3D	U-Net U-Net	Template-based data augmentation, N4 bias correction, denoising	65/35 77/23	DSC (HP gas)=0.92 DSC (Proton) = 0.94
*Akila Agnes et al. (2018*)^31^	LDCT	Whole lung	NR	220	2D	CDWN	Normalised [mean = 0]	91/9	DSC = 0.95 ± 0.03 JSC = 0.91 ± 0.04
*Zha et al. (2019*)^46^	UTE proton MRI	Whole lung	Healthy, CF, asthma	45	2D	CED (U-Net and autoencoder)	Denoising, bias field correction, body mask	5-fold CV	DSC (right) = 0.97±0.015 DSC (left) = 0.96±0.012
*Hwang & Park (2017*)^77^	X-ray	Whole lung	Healthy, lung nodules	247	2D	U-Net		2-fold CV	DSC = 0.980±0.008 JSC = 0.961±0.015 ASD (mm) = 0.675±0.122 ACD (mm) = 1.237±0.702
*Souza et al. (2019*)^78^	X-ray	Whole lung	Healthy, Tuberculosis	138	2D	ResNet-18 with FC layer	Scaled to same input size, post processing erosion, dilation, filtering	73/27	DSC = 0.936 JSC = 0.881
*Dai et al. (2018*)^64^	X-ray	Whole lung	Healthy, Tuberculosis, lung nodules	385	2D	SCAN (structure correcting adversieral network)	Scaled to same input size	85/15	IoU = 94.7±0.4% DSC = 0. 973 ± 0.02
*C. Wang (2017*)^79^	X-ray	Whole lung	Healthy, lung nodules	247	2D	Multi task U-Net	Scaled to same input size, post processing hole filling	NR	JSC = 0.959 ± 0.017 AD = 1.29 ± 0.80 mm
*Novikov et al. (2018*)^32^	X-ray	Whole lung	Healthy, lung nodules	247	2D	InvertedNet + All-dropout	Normalised [mean = 0, SD = 0]	3-fold CV	DSC = 0.974 JSC = 0.949
*Hooda et al. (2018*)^50^	X-ray	Whole lung	Healthy, Tuberculosis, lung nodules	385	2D	FCN-8+dropout	Scaled to same input size, random cropping	75/25	DSC = 0.959
*Mittal et al. (2018*)^51^	X-ray	Whole lung	Healthy, Tuberculosis, lung nodules	385	2D	LF-SegNet	Scaled to same input size, random cropping	48/52	DSC = 0.951
*Gaal et al. (2020*)^33^	X-ray	Whole lung	Healthy, Tuberculosis, lung nodules	1047	2D	Adversarial attention U-Net	Scaled to same input size, CLAHE, Normalisation [−1,1]	24/76	DSC = 0.962±0.04
*Chen et al. (2019*)^80^	CT	Lung tumour	Lung cancer	134	3D	HSN (2*D* + 3D CNN)		78/22	DSC = 0.888±0.033
*Jiang et al. (2018*)^11^	CT, MRI	Lung tumour	Lung cancer	400 CT (377) MRI (23)	2D	Tumour aware semi-supervised Cycle-GAN	Scaled to same input size, Image synthesis from CT to MRI, body mask	98/2	DSC = 0.63 ± 0.24 HD95 = 11.65±6.53
*Jiang et al. (2019*)^17^	CT, MRI	Lung tumour	Lung cancer	405 CT (377) MRI (28)	2D	Tumour aware pseudo MR and T2w MR U-Net	Scaled to same input size, Image synthesis from CT to MR, Clipped [−1000,500 HU] and [0,667], Normalised [−1, 1]	95/5	DSC = 0.75±0.12 HD95 = 9.36±6.00 mm VR = 0.19±0.15
*Tahmasebi et al. (2018*)^18^	MRI	Lung tumour	Lung cancer	6	2D	Adapted FCN	Rescaled 10–95% of intensities, Normalisation [0,1]	5-fold CV	DSC = 0.91 ± 0.03 HD = 2.88 ± 0.86 mm RMSE = 1.20 ± 0.34
*Z. Zhong et al. (2019*)^19^	FDG PET, CT	Lung tumour	Lung cancer	60 PET (60) CT (60)	3D	DFCN Co-Seg U-Net	Scaled to same input size, Clipped [−500,200 HU] and [0.01,20]	80/20	DSC (CT) = 0.861±0.037 DSC (PET) = 0.828±0.087
*Zhao et al. (2019*)^52^	PET, CT	Lung tumour	Lung cancer	84 PET (84) CT (84)	3D	V-Net +feature fusion	Cropped to ROI	57/43	DSC = 0.85±0.08 VE = 0.15±0.14
*Zhou et al. (2019*)^20^	CT	Lung tumour	NR	1350	3D	P-SiBA	Transfer learning from ImageNet ILSVRC‐2014, Cropped to ROI, Rescaled by +1000 HU and dividing by 3000 and Normalisation [0,1]	NR	DSC = 0.809 ± 0.12 HD = 7.612 ± 5.03 mm *vs* = 0.883 ± 0.13
*Moriya et al. (2018*)^53^	Micro CT	Lung tumour	Lung cancer	3	3D	JULE CNN + k-means	Body mask, patch extraction		NMI = 0.390
*Imran et al. (2019*)^81^	CT	Lobes	COPD, ILD	563	3D	Progressive dense V-Net		48/52	DSC (*n* = 84)=0.939±0.02 DSC (*n* = 154)=0.950±0.007 DSC (*n* = 55)=0.934
*Park et al. (2019*)^21^	CT	Lobes	COPD	196	3D	U-Net	Clipped [-1024,–400 HU]	80/20	DSC = 0.956 ± 0.022 JSC = 0.917 ± 0.031 MSD = 1.315 ± 0.563 HSD = 27.89±7.50
*Wang et al. (2018*)^13^	CT	Lobes	COPD, IPF	1280	3D	DenseNet	Clipped −1000 to +1000 HU, Normalisation [0,1]	5-fold CV	DSC = 0.959±0.087 ASD = 0.873±0.61 mm
*Hatamizadeh et al. (2019*)^34^	CT	Lung lesion	NR	87	3D	DALS CNN	Scaled to same input size, Normalisation [NR]	90/10	DSC = 0.869 ± 0.113 HD = 2.095 ± 0.623 mm
*Kalinovsky et al. (2017*)^54^	CT	Lung lesion	Tuberculosis	338	2D	GoogLeNet CNN	Images cropped into four quadrants	80/20	IoU = 0.95 ROC = 0.775
*Gerard et al. (2019*)^22^	CT	Lung fissure	COPD, Lung cancer	5327	3D	Two Seg3DNets	Clipped [-1024,–200 HU], Linear rescaling	30/70	ASD = 1.25 SDSD = 2.87
*Sandkühler et al. (2019*)^35^	MRI	Lung defect region	NR	35	2D	GAE-LAE RNN with LCI Loss	Z-normalisation [−4,4], Lung mask, Normalisation [0,1], Histogram stretching	80/20	Qualitative evaluation - 42% images rated ‘very good’, 19% rated ‘perfect’
*Vakalopoulou et al. (2018*)^82^	CT	ILD pattern	ILD	46	2D	AtlasNet		37/63	DSC = 0.677 HD = 3.981 mm ASD = 1.274 mm
*Anthimopoulos et al. (2019*)^55^	CT	ILD pattern	ILD	172	2D	FCN-CNN	Pre-computed lung mask	5-fold CV	Accuracy = 81.8%
*B. Park et al. (2019*)^83^	CT	ILD pattern	COP, UIP, NSIP	647	2D	U-Net		88/12	DSC = 0. 988 ± 0.006 JSC = 0.978 ± 0.011 MSD = 0.27 ± 0.18 mm HSD = 25.47 ± 13.63 mm
*Gao et al. (2016*)^56^	CT	ILD pattern	ILD	17	2D	CNN based CRF unary classifier	Transfer learning from ImageNet, Pre-computed lung mask		Accuracy = 92.8%
*Suzuki et al. (2020*)^84^	CT	Diffuse lung disease	NR	372	3D	U-Net		5-fold CV	DSC = 0.780±0.169
*Wang et al. (2018*)^85^	MRI	Foetal lung	NR	18	2D	BIFSeg P-Net	Trained on different organs, Image specific fine-tuning	66/33	DSC = 0.854±0.059
*Rajchl et al. (2017*)^36^	MRI	Foetal lung	Healthy, IUGR	55	3D	DeepCut CNN + CRF	Bounding box for ROI, Bias correction, Normalisation [mean = 0], Transfer learning from LeNet	5-fold CV	DSC = 0.749±0.067
*Edmunds et al. (2019*)^86^	Cone-beam CT	Diaphragm	Lung cancer	10	2D	Mask R-CNN	Scaled to same input size	9-fold CV	Mean error = 4.4 mm
*C. Wang et al. (2019*)^57^	CT	Airways	NR	38	3D	Spatial-CNN (U-Net)	Random cropping	92/8 3-fold MCCV	DSC = 0. 887 ± 0.012 CO = 0.766 ± 0.06
*Juarez et al. (2019*)^58^	CT	Airways	Lung cancer	32	3D	U-Net GNN	Bounding box for ROI	63/37	DSC = 0.885 Airway completeness = 74%
*Yun et al. (2019*)^23^	CT	Airways	COPD	89	2D	2.5D CNN	Clipped [−700,700 HU]	78/22	Mean Branch detected = 65.7%
*Juarez et al. (2018*)^59^	CT	Airways	Healthy, CF, CVID	24	3D	U-Net	Bounding box for ROI	75/25	DSC = 0.8

ACD, Average contour distance; AD, Average distance; ASD, Average surface distance; CDWN, Convolutional deep wide network; CE, Classification error; CF, Cystic fibrosis; CLAHE, Contrast limited adaptive histogram equalisation; CNN, Convolutional neural network; CO, Centreline overlap; COPD, Chronic obstructive pulmonary disorder; CV, Cross-validation; CVID, Common variable immunodeficiency disorders; DSC, Dice similarity coefficient; FDG, Fluorine-18‐fluorodeoxyglucose; GAN, Generative adversarial network; HD95, Hausdorff distance 95%; HD, Hausdorff distance; HSD, Hausdorff surface distance; HU, Hounsfield unit; ILD, Interstitial lung disease; IPF, Idiopathic pulmonary fibrosis; IUGR, Intrauterine growth restriction; IoU, Intersection over union; JSC, Jaccard similarity coefficient; LOOCV, Leave-one-out cross-validation; MAP, Mean average precision; MCCV, Monte carlo cross-validation; MSD, Mean surface distance; NMI, Normalised mutual information; NR, Not reported; NSIP, Nonspecific interstitial pneumonia; PVD, Percent ventilated defect; RMSE, Root mean square error; ROC, Receiver operating characteristic; ROI, Region of interest; SD, Standard deviation; SDSD, Standard deviation of surface distances; UIP, Usual interstitial pneumonia; VE, Volume error; VR, Relative volume ratio; VS, Volumetric similarity.

The entries are arranged alphabetically by pulmonary ROI, followed by modality.

a The training data set includes internal validation data.

### CT segmentation

CT is the most common modality for clinical lung imaging due to superior spatial resolution, rapid scan times and widespread availability. This is reflected in the DL lung segmentation literature with the majority of studies to date focusing on CT. For whole-lung segmentation, 3D networks are often used, whereas in interstitial lung disease (ILD) pattern segmentation, only 2D networks have been applied to date. The application often dictates the use of 2D and 3D networks; segmentation of the whole lung leads to a volumetric 3D region in which features such as overall lung shape, or the position of the trachea can be encoded. In contrast, segmenting ILD patterns is often conducted on central 2D slices; hence, a 2D network may be more appropriate as, in this approach, no features are conserved between slices.^55,83^

Across the CT papers reviewed, both the median and mode training/testing data splits were 80/20%, with many using k-fold cross-validation with less than 50 patients. Even as an independent testing set, using only 5–10 patients for testing limits generalisability. Moreover, some studies cite the number of images or 2D slices rather than the number of subjects. If data from the same subject are included in both the testing and training phases, it is likely that the algorithm has already seen a similar slice from the same patient as the individual data points are spatially correlated and do not strictly represent independent data points.

The Dice similarity coefficient (DSC) overlap metric is the most common evaluation metric used. Most studies tackling whole-lung segmentation report DSC values above 0.90, with some achieving values above 0.98. For other pulmonary ROIs, the highest DSC values reported are often lower (*e.g.* DSC (airways) ≈ 0.85). However, overlap metrics such as the DSC can be insensitive to errors in large volumes as the percent error is low compared to the overall pixel count.^87^ Frequently, high DSC values are reported despite errors that require significant manual intervention before a segmentation is clinically useful. As the airways occupy smaller volumes, the DSC metric is more sensitive. In terms of Hausdorff-based distance metrics, whole-lung segmentation studies report HD95 values ≈10 mm; however, Dong et al^70^ report a HD95 as low as 2.249 ± 1.082 mm averaged across both lungs. The lack of a standardised evaluation metric can make direct comparisons between different methods challenging.

Image segmentation is challenging to evaluate. Currently, manual segmentations by expert observers are used as the gold-standard; however, it is well-known that expert segmentations are susceptible to interobserver variability.^88^ Often, only one observer segments all the images in a training data set; hence, if a different observer segments the testing images, the algorithm may not perform as expected. This poses problems for widespread generalisation if certain biases in segmentation are preserved as there is no clear ‘true’ expert segmentation; therefore, differences in DL segmentations and expert segmentations may not be solely the result of DL errors. Most expert segmentations are conducted using semi-automatic software and image editing tools; the tools given to the user can convey a propensity for features, such as smooth lung borders, which may, in fact, be inaccurate. In other anatomical sites such as the liver, a DSC of 0.95 was obtained by DL; the interobserver variability for the DL approach was 0.69% compared to 2.75% for manual expert observers.^89^ The low degree of interobserver variability in DL segmentations may be a positive step towards consistent segmentations between institutions. Using multiple expert segmentations and averaging the error may reduce interobserver variability effects; however, this is unlikely to be widely adopted due to the time required. In addition, medical imaging grand challenges can provide diverse data from multiple institutions with corresponding expert segmentations, limiting the extent of individual researcher bias.

### MRI segmentation

**Figure 9.**
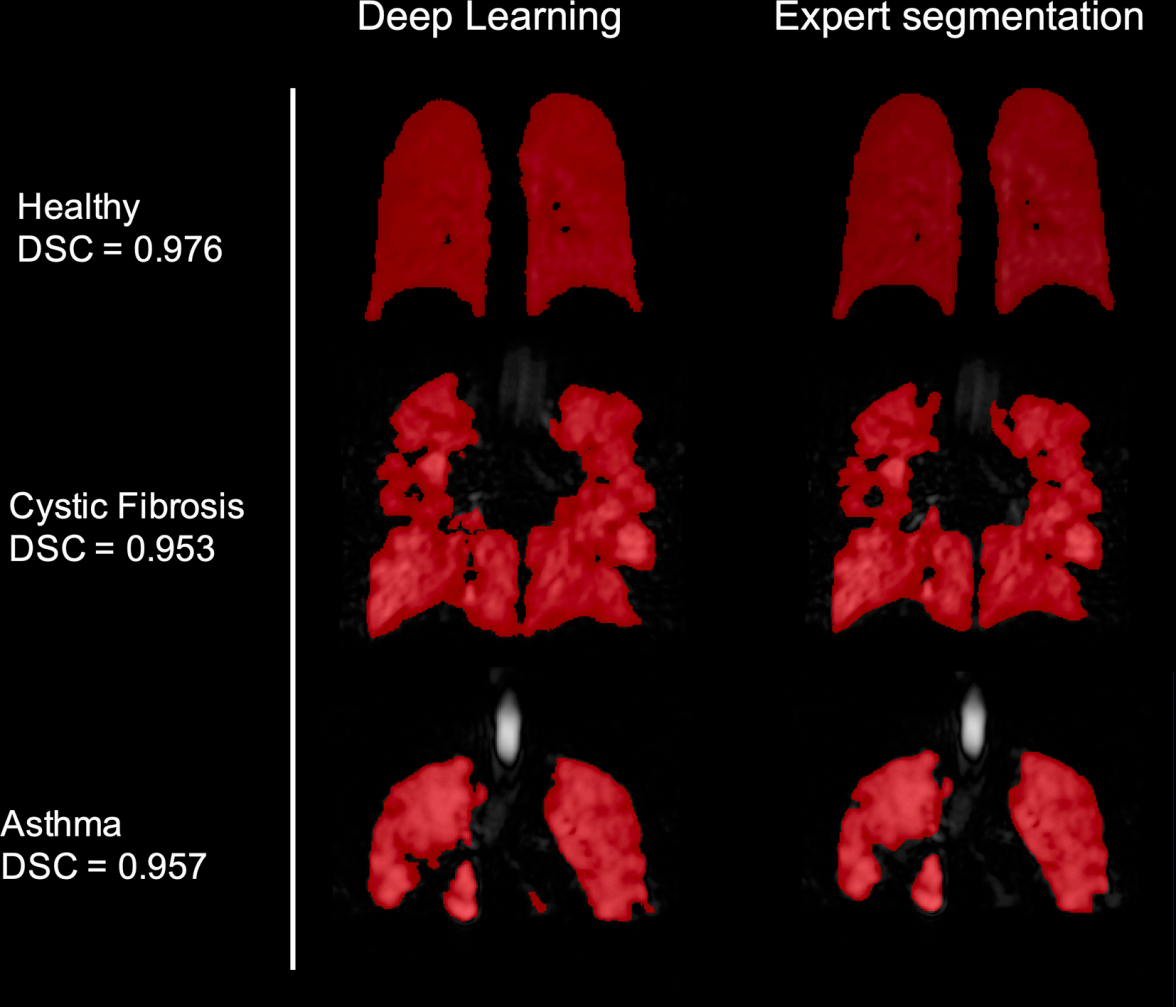
Example images from the authors’ own work using deep learning for hyperpolarised gas MRI segmentation. The ^129^Xe MR ventilation images are taken from three subjects in a testing set, a healthy volunteer, asthma patient and cystic fibrosis patient. The patient images selected are characterised by significant ventilation defects. These are compared to expert segmentations of the same image. DSC values are displayed for all images. DSC, Dice similarity coefficient.

There are limited studies to date regarding pulmonary MRI segmentation, attributable perhaps to less widespread clinical use of the modality and lack of large-scale annotated pulmonary MRI data sets. However, pulmonary MRI techniques, such as contrast-enhanced lung perfusion MRI and hyperpolarised gas ventilation MRI, can provide further insights into pulmonary pathologies currently not possible with alternative techniques.^90^ Quantitative biomarkers derived from hyperpolarised gas MRI, including the ventilated defect percentage, require accurate segmentation of ventilated and whole-lung volumes which can be very time consuming when performed manually. Example images of DL-based hyperpolarised gas MRI segmentations are provided in Figure 9.

Tustison et al^47^ used CNNs to provide fast, accurate segmentations for hyperpolarised gas and proton MRI.^47^ A 2D U-Net was used for hyperpolarised gas MRI segmentation whilst a 3D U-Net was used for proton MRI segmentation. They introduced a novel template-based data augmentation method to expand the limited lung imaging data. Hyperpolarised gas and proton MR images were segmented with DSC values of 0.94 ± 0.03 and 0.94 ± 0.02, respectively. Zha et al evaluated DL-based proton MRI segmentation, which yielded an average DSC of 0.965 across both lungs, outperforming conventional region growing and k-means techniques.^46^

### X-ray segmentation

Although the majority of segmentation studies reviewed used CT and MRI, early studies focused on X-ray segmentation.^77,79^ This was due to the public availability of large-scale, annotated X-ray datasets, such as the Japanese Society of Radiological Technology (JSRT)^91^ and Montgomery^92^ data sets, enabling researchers to experiment with large numbers of images not previously accessible. The majority of X-ray studies reviewed used these datasets, making comparisons between methods more applicable.^32,50,51,64,78,79^

### Registration

Image registration is the process of transforming a moving image onto the spatial domain of a fixed image. Registration is used in numerous applications within the lung imaging field, including adaptive radiotherapy,^93^ computation of functional lung metrics such as the VDP^94^ and generation of surrogates of regional lung function from multi-inflation CT^95^ or ^1^H MRI.^96^

However, most image registration algorithms assume that the moving and fixed images’ topology are the same. This is not always the case in lung imaging as often functional images do not follow the same topology as structural images, especially in individuals with severe pathologies where functional lung images may show substantial heterogeneity.^97^ Studies describing DL-based pulmonary registration applications are summarised in Table 3.

**Table 3.** Summary of reviewed studies using deep learning for lung image registration

*Study*	*Modality*	*Disease*	*Public data set*	*Number of subjects*	*Dimensionality*	*Architecture*	*Preprocessing*	*Percentage data split* (training*/testing)	*Performance*
*Eppenhof et al. (2018*)^98^	4DCT	Lung cancer	DIR-LAB, CREATIS	17	3D	Modified VGG	Synthetic DVFs for data augmentation	42 (CREATIS) / 58 (DIR-LAB)	TRE = 4.02±3.08
*Eppenhof & Pluim (2019*)^24^	4DCT	Lung cancer	DIR-LAB, CREATIS	17	3D	Modified U-Net	Synthetic DVFs for data augmentation, Resized, Pre-computed body mask, intensity-based lung mask < −250 HU	42 (CREATIS) / 58 (DIR-LAB)	TRE = 2.17±1.89 mm
*Ali & Rittscher (2019*)^99^	4DCT	Lung cancer	DIR-LAB, CREATIS	17	2D	Conv2Wrap (Linear and Deformable ConvNet)		58 (DIR-LAB) / 42 (CREATIS)	DSC = 0.90 JSC = 0.84
*Sentker et al. (2018*)^37^	4DCT	Lung cancer	DIR-LAB, CREATIS	86	3D	GDL-FIRE^4D^ U-Net with VarReg	Normalisation [0,1], Cropped to same input size, Pre-computed body mask	69/31 (DIR-LAB, CREATIS, In house)	TRE (DIR-LAB) = 2.50±1.16 mm TRE (CREATIS) = 1.74±0.57 mm
*Fletcher and Baltas (2020*)^38^	4DCT	Lung cancer	DIR-LAB, CREATIS, Sunnybrook	31	3D	U-Net one-shot learning	Pre-computed body mask, Normalisation [mean = 0, SD = 1]	LOOCV (DIR-LAB) 0/100 (CREATIS)	TRE (DIR-LAB) = 1.83±2.35 mm TRE (CREATIS) = 1.49±1.59 mm
*Fu et al. (2020*)^25^	4DCT	Lung cancer	DIR-LAB	20	3D	LungRegNet (CourseNet, FineNet)	Vessel enhancement, Clipped at −700 HU	5-fold CV, DIR-LAB testing	MAE (in house)=52.1±18.4 TRE (in house)=1.00±0.53 TRE (DIR-LAB) = 1.59±1.58 mm
*Jiang et al. (2020*)^26^	4DCT	Lung cancer	DIR-LAB, SPARE	32	3D	MJ-CNN	Clipped [-1000,–200 HU], Normalisation [0,0.2]	75 (SPARE, DIR-LAB) / 25 (DIR-LAB)	TRE = 1.58±1.19 mm
*De Vos et al.(2019*)^27^	4DCT, CT	Lung cancer	DIR-LAB, NLST	2070	3D	DLIR framework ConvNet	Clipped [-1000,–200 HU], Normalisation [0,1]	99 (NLST) / 1 (NLST, DIR-LAB)	DSC (NLST) = 0.75±0.08 HD (NLST) = 19.34±13.41 TRE (DIR-LAB) = 5.12±4.64 mm
*Sokooti et al. (2017*)^100^	CT	COPD		19	3D	RegNet CNN	Synthetic DVFs for data augmentation, Initial affine registration	63/37 (SPREAD)	TRE = 4.39 ± 7.54 mm
*Sokooti et al. (2019*)^101^	CT, 4DCT	Lung cancer, COPD	SPREAD, DIR-LAB	39	3D	RegNet CNN (U-Net)	Synthetic DVFs for data augmentation, Initial affine registration	54 (SPREAD, DIR-LAB COPD) / 46 (SPREAD, DIR-LAB)	TRE (DIR-LAB) = 1.86±2.12 mm
*Blendowski & Heinrich (2019*)^60^	CT	COPD	DIR-LAB	10	3D	CNN	Cropped to lung region	LOOCV (DIR-LAB)	TRE = 3.00 ± 0.48 mm
*Qin et al. (2019*)^102^	CT, MRI	COPD	COPDGene	1000	2D	UMDIR-LaGAN	Cross-modality registration, transformation into domain invariant latent space	90/10 (COPDGene)	DSC = 0.967±0.03 HD = 8.257±4.43 mm MCD = 0.71±0.44 mm
*Galib et al. (2019*)^39^	CT, CBCT	Healthy, COPD, Lung cancer	DIR-LAB, VCU	27	3D	CNN	Normalisation [0,1]	37 (DIR-LAB) / 63(VCU)	AUC-ROC = 0.882±0.11 CI=68%
*Ferrante et al. (2018*)^40^	X-ray	Healthy, Lung nodule	JSRT	247	2D	U-Net	Normalisation [0–1], Domain adaption Cardiac MR	81/19 (JSRT)	MAD ≈ 6.3 CMD ≈ 5 mm DSC ≈ 0.9
*Mahapatra et al. (2018*)^103^	X-ray	Multiple	NIH-ChestXray14	420	2D	JRSNet (cycleGAN with U-Net)	Joint segmentation and registration	NR (SCR, NIH-ChestXray14)	TRE = 7.75 mm
*Stergios et al. (2018*)^28^	MRI	Systemic sclerosis, healthy		41	3D	CNN with transformation layer	Clipped [0, 1300], Normalisation [0,1]	68/32	DSC = 0. 915 ± 2.33 Euclydian error = 4.358 mm

AUR-ROC, Area under curve-receiver operator characteristic; CMD, Contour mean distance; CNN, Convolutional neural network; COPD, Chronic obstructive pulmonary disorder; CV, Cross-validation; DLIR, Deep learning image registration; DSC, Dice similarity coefficient; HD, Hausdorff distance; HU, Hounsfield unit; JSC, Jaccard similarity coefficient; LOOCV, Leave-one-out cross-validation; MAD, Mean absolute differences; MAE, Mean absolute error; MCD, Mean contour distance; MRF, Markovian random field; TRE, Target registration error; VGG, Visual geometry group.

Eppenhof and Pluim^24^ built upon previous work by Lafarge et al^98^ using publicly available data sets to directly map displacement vector fields from inspiratory and expiratory CT pairs using a 3D U-Net with extensive data augmentation. Synthetic transforms were used to directly train the network as the deformation fields are known. The approach achieved fast, accurate registrations, reducing mean TRE from 8.46 to 2.17 mm. The results are further validated using landmarks from multiple observers, indicating the level of interobserver variability. Notwithstanding, only 24 images for testing and training were used, limiting the study’s generalisability. In addition, synthetic transforms do not directly represent real transforms likely found in patients.

Other approaches use a CNN to learn expressive local binary descriptors from landmarks before applying Markov random field registration.^60^ This is compared to a method using handcrafted local descriptors with high self-similarity, facilitating faster computation. The results suggest that a combination of both CNN-learned descriptors and handcrafted features produce the best registration results.

In a generic registration approach, a U-Net-like architecture with a differentiable spatial transformer that can register both X-ray and MR images was used.^40^ The algorithm was evaluated using the contour mean distance (CMD). CMD was approximately 5 mm on average across the testing data. Whilst this is a less accurate registration than other methods reviewed, it is more broadly applicable; the generic algorithm (in this case trained on X-ray and MR images) can learn features that are independent of modality. By fixing these weights and adding additional layers, transfer learning can then be applied to a specific modality; the additional data across modalities may lead to improved results.^104^

### Reconstruction

Image reconstruction is the process of generating a usable image from the raw data acquired by a scanner. CT and SPECT reconstruction fundamentally differ from MRI reconstruction and, as such, the role of DL in these applications is also different. CT and SPECT reconstruction use analytic (*e.g.* filtered backprojection) or iterative algorithms to produce 3D images from projections taken at multiple angles around a subject. MRI reconstruction, in contrast, produces images by transforming raw k-space data via Fourier transforms. Full details of image reconstruction methods have been described elsewhere.^105,106^ Studies describing DL-based lung image reconstruction applications are summarised in Table 4.

**Table 4.** Summary of reviewed studies using deep learning for lung image reconstruction

*Study*	*Modality*	*Disease*	*Number of patients*	*Dimensionality*	*Architecture*	*Preprocessing*	*Percentage data split* (training*/testing)	*Performance*
*Beaudry et al. (2019*)^41^	4D cone beam CT	Lung cancer	16	2D	Sino-Net (Modified U-Net)	Cropped to same input size, Sinogram Normalisation [0,1]	88/12	RMSE Translational = 1.67 mm (other metrics given)
*Lee et al. (2019*)^107^	CT	COPD	60	2D	FCN	No sinogram used	Dataset 1: 80/20 Dataset 2: 40/60	Mean reduction RMSE (Dataset 1) = 65.7±15.8% Mean reduction RMSE (Dataset 2) = 59.6±5.5%
*Ge et al. (2020*)^108^	CT	Liver lesion	5413	2D	ADAPTIVE-NET CNN	Convert from HU to linear attenuation coefficient	90/10	PSNR = 43.15±1.9 SSIM = 0.968±0.013 Normalized RMSE = 0.0071±0.002
*Duan et al. (2019*)^42^	HP Gas MRI	COPD, nodule, PTB, healthy, asthma	72	2D	C-Net and F-Net (U-Net based)	Under sampled K-space (AF = 4), Removed SNR below 6.6, Normalisation [0,1]	NR	MAE = 4.35% SSIM = 0.7558 VDP bias = 0.01±0.91%
*Dietze et al. (2019*)^109^	^99^mTc-MAA SPECT	Liver Cancer	128	2D	CNN	Initial filtered back projection	94/6	LSF = 5.1% CNR = 12.5

CNN, Convolutional neural network; CNR, Contrast to noise ratio; COPD, Chronic obstructive pulmonary disorder; EIT, Electrical impedance tomography; HU, Hounsfield unit; LSF, Lung shunting fraction; MAE, Mean absolute error; PSNR, Peak signal to noise ratio; PTB, Pulmonary tuberculosis; RMSE, Root mean square error; SSIM, Structural similarity index metric; VDP, Ventilation defect percentage; VDP, Volume defect percentage; 99mTc-MAA, Technetium-99m macroaggregated albumin.

a The training data set includes internal validation data

CT/SPECT images can be reconstructed accurately using Monte-Carlo-based iterative reconstruction^110^; however, this process is computationally expensive and time-consuming.^111^ In addition, multiple studies have demonstrated the success of analytical methods such as filtered backprojection.^105^ Building upon this, CNNs have been used to speed up the process of filtered backprojection to shorten reconstruction times.^109^ The results suggest DL can accurately reconstruct SPECT images in under 10 sec. Furthermore, the authors compare clinical metrics, such as the lung shunting fraction (LSF), between methods in a specific time frame. DL produced an LSF of 4.7% comparable to 5.8% for Monte-Carlo methods, indicating the potential for use in clinical applications.^109^

Multiple studies have employed DL for MRI reconstruction^112^ but only one published study has applied it to pulmonary MRI.^42^ MRI of the lungs can take upwards of 10 sec to acquire, often requiring that patients maintain inflation levels for a significant period; this can be particularly challenging for patients with severe lung pathologies. Compressed sensing can be used to reconstruct randomly undersampled k-space in conjunction with regularisation methods to produce accurate reconstructions in hyperpolarised gas MRI^113,114^ and enables reduced acquisition time without significantly reducing image quality. A coarse-to-fine neural network has been proposed to yield an accurate hyperpolarised gas MRI scan with an accelerating factor of 8 (undersampled 1/8 of k-space).^42^ The method can also improve inherent spatial coregistration accuracy when acquiring proton and hyperpolarised gas MRI in the same breath,^115^ possibly alleviating the need for substantial post-acquisition image registration.

Tangentially related to the goal of image reconstruction, images can also be improved further using image enhancement at the post-acquisition stage. Multiple studies have shown the effectiveness of using CNNs combined with gradient regularisation and superresolution modules to enhance low-dose CT images with noise and artefacts, potentially limiting radiation exposure without degrading image quality.^116,117^

### Synthesis

Image synthesis, also referred to as regression, is the process of generating artificial images of unknown target images from given source images. Synthesis has been applied to a range of applications, such as generating functional or metabolic images from structural images. For example, estimating contrast-based functional images from routinely acquired non-contrast structural modalities reduces the need for additional scans, specialised equipment and administration of contrast agents. Even within traditional model-based techniques, accurate synthesis has proved challenging due to the complex mathematical functions mapping input to output images. The development of DL architectures such as GANs enables a more unsupervised approach, which lends itself to the complex problem of synthesis.^9^ Studies describing DL-based lung image synthesis applications are summarised in Table 5.

**Table 5.** Summary of reviewed studies using deep learning for lung image synthesis

*Study*	*Modality* (*original* ⇒ *target*)	*Disease*	*Number of subjects*	*Dimensionality*	*Model*	*Preprocessing*	*Percentage data split* (training*/testing)	*Performance*
*Bi et al. (2017*)^118^	CT ⇒ FDG PET	Lung cancer	50	2D	Multichannel-GAN (U-Net)	Manual segmentation of tumour/lymph nodes, axial slices containing tumours only	50/50	MAE = 4.6 PSNR = 28.06
*Jang et al. (2019*)^119^	CT ⇒^99^mTc-MAA SPECT perfusion	Lung cancer	54	2D	Conditional GAN	Resized images, segmentation and removal of bone, soft tissue and heart	91/9	MS-SSIM = 0.87 γ index 2%/2mm = 97.7±1.2%
*Zhong et al. (2019*)^61^	4DCT ⇒ CT ventilation	Lung cancer, COPD	82	2D	Deep CNN	Images cropped to ROI	10-fold CV	MSE = 7.6% γ index 5%/5mm = 80.6±1.4% SSIM = 0.880±0.035
*Liu et al. (2020*)^43^	4DCT ⇒^99^mTc-Technegas SPECT ventilation	Lung cancer, oesophageal cancer	50	2D	U-Net	Pre-computed lung mask, normalisation [0,1], post-processing normalisation [90^th^ percentile]	10-fold CV	Spearman’s *ρ* = 0.73±0.17 DSC = 0.73±0.09
*Ren et al. (2019*)^29^	CT ⇒^99^mTc-MAA SPECT perfusion	Lung cancer	30	3D	U-Net	Clipped [-1000,–300 HU] for segmentation, normalisation [0,1]	83/17	Correlation coefficient = 0.53 ± 0.14
*Preiswerk et al. (2018*)^120^	Ultrasound ⇒ MRI	NR	7	3D	LRCN	PCA = 10 components	66/33 (conducted in time segments)	SSE = 39.0 ± 12
*Olberg et al. (2018*)^44^	MRI ⇒ CT	NR	41	NR	GAN (U-Net)	Normalisation [NR], pre-computed body mask	90/10	3D γ index passing rate 99.2% Lung V20% difference = 0.11%

CNN, Convolutional neural network; COPD, Chronic obstructive pulmonary disease; FDG, Fluorine-18‐fluorodeoxyglucose; GAN, Generative adversarial network; HU, Hounsfield unit; LRCN, Long-term recurrent convolutional network; MAE, Mean absolute error; MSE, Mean square error; MS-SSIM, Multi-scale structural similarity index metric; NR, Not reported; PCA, Principle component analysis; PSNR, Peak signal to noise ratio; ROI, Region of interest; SSE, Sum of squared error; 99mTc-MAA, Technetium-99m macroaggregated albumin.

DL has been used to generate synthetic fluorine-18‐fludeoxyglucose (FDG) PET images from CT images via a GAN.^118^ The GAN’s inputs were varied to include either a CT image, label, or both CT and corresponding label; the multichannelled GANs (M-GAN) provided the most accurate synthetic PET images, demonstrating that multiple inputs increase synthesis accuracy. To explore this further, the authors also evaluate the synthetic PET images by feeding them into a network as training data. The network aims to delineate tumours by learning relationships from the training data; the data were then divided into real PET images and synthetic PET images. The trained model was then evaluated on unseen tumour detection problems. The synthetic PET-trained network produced 2.79% lower recall accuracy. This indicates that, as a whole, the synthetic PET images are closely related to the real images in terms of tumour identification. The paper posits that synthetic PET images can be used as additional training data in other DL tasks. However, it is unclear if synthetic PET images can be used in treatment planning and other clinical tasks with this level of accuracy.^118^

GANs have continued to show promise in synthesis problems.^119^ CT images have been used to generate SPECT images via a conditional GAN (cGAN) instead of a CNN.^29^ The method used a 2D GAN with 49 patients consisting of 3054 2D images as training data; the testing data contains 5 patients. cGANs differ from the regular GAN architecture by using both the observed image and a random noise vector, mapping these to the output image instead of only the noise vector. The generator used is based on the U-Net architecture with multiple inputs. Synthetic and real SPECT images were compared using the multiscale structural similarity index measure (MS-SSIM), yielding MS-SSIM = 0.87. Further analysis used a γ index with a passing rate of 97.7±1.2% with 2%/2 mm. The authors note qualitatively that errors occur more frequently at the base of the lungs, possibly caused by the increased deformation in this region. A key limitation for synthesis methods is the errors introduced by the registration of source and target images. Consequently, it has been suggested that images that are not matched anatomically due to breathing discrepancies are excluded,^119^ complicating validation for clinical adoption.^29,119^

A major application of DL image synthesis is for MR-guided radiotherapy. The current paradigm in radiotherapy is to derive electron density information required for dose calculations directly from CT scans; MRI does not directly provide this information. DL has been invoked to generate pseudo-CT images for use in MR-guided stereotactic body radiotherapy using GANs, precluding the need for CT.^44^

Zhong et al used a CNN to synthesise ventilation images from 4DCT scans.^61^ Whilst good performance was observed, the major limitation of this study is that the target images in the training phase were CT-based surrogates of ventilation generated from aligned inspiratory and expiratory CT scans via deformable registration and computational modelling. These images are still the subject of intense validation efforts.^121^ Using more direct measures of regional lung function, such as hyperpolarised gas MRI, and larger data sets are critical to the success of future work in structure-to-function DL synthesis applications.

## Future research directions

The studies reviewed show that DL has significant potential to outperform more traditional methods in a wide range of lung image analysis applications. Novel ways of using DL to synthesise more training examples^122^ or combine segmentation and registration in one process^103^ have been shown to enhance performance. The scope of such innovation is still in its infancy, providing an opportunity for novel technical developments.

As shown through the improved performance observed by combining traditional approaches with machine learning and DL for registration, great synergy can be achieved by combining DL and conventional image processing approaches.^60^

In image synthesis, researchers have developed techniques to synthesise CT images from MRI scans of the brain^123^; similar advancements in lung imaging would allow patients to receive less radiation exposure as well as reduce the cost and time for additional scans. Using synthesis to generate functional lung images from routinely acquired structural images would allow clinicians to understand which areas of the lungs are ventilated or perfused without the need to acquire dedicated functional scans, which often require contrast agents and specialised equipment, reducing costs and acquisition times. Such applications require further DL research in architectural development and the input of lung imaging experts. Using DL for CT enhancement to reduce radiation dose or improve compressed sensing methods in MRI has the potential to reduce scan times, improving image quality and patient compliance.

Promising results have been shown for both proton MRI and hyperpolarised gas MRI segmentation^47^; however, further work is required to demonstrate accurate MRI segmentation in an independent multicentre validation. The importance of collaborative research to boost training data and inject heterogeneity of centre and scanner will lead to more robust and generalisable models. The paucity of published DL studies in functional lung imaging (only 12.9% of reviewed studies here) provides significant opportunities for innovations and further research in this field.

The literature on CT segmentation provides a positive picture of the success of DL methods in providing fast, accurate automatic segmentations. However, producing impressive results in a research setting is no substitute for clinical validation. Long-term clinical case studies are required with large numbers of patients before these novel developments have a real impact. The ‘black box’ nature of DL methods and the lack of explainability of generated outputs can undermine clinicians and patients’ trust, despite, or even because of, an unprecedented level of hype. Another challenge is transparency; although most software used for DL is well documented and open source, a requirement for continued use, the open-source nature also generates safety concerns relating to software edits and bugs. Developing a standardised literature consensus on validation and evaluation procedures is key to ensuring transparency. All of these challenges need to be overcome before DL can live up to its full potential.

## Conclusions

We have reviewed the role of DL for several lung image analysis tasks, including segmentation, registration, reconstruction and synthesis. CT-based lung segmentation was the most prevalent application where exceptional performance has been demonstrated. However, research in other applications and modalities, including functional lung imaging, is still in its infancy. A concerted effort from the research community is required to develop the field further. Before widespread clinical adoption is achievable, challenges remain concerning validation strategies, transparency and trust.
